# Epstein-Barr-Virus-Related Lymphoproliferative Disorder in a Patient With Primary Myelofibrosis: A Case Report and Literature Review

**DOI:** 10.7759/cureus.56586

**Published:** 2024-03-20

**Authors:** Seigi Oshima, Shojiro Inano, Toshiyuki Kitano

**Affiliations:** 1 Hematology, Kitano Hospital Medical Research Institute, Osaka, JPN; 2 Hematology and Oncology, Kyoto University Hospital, Kyoto, JPN

**Keywords:** diffuse large b cell lymphoma (dlbcl), myeloproliferative neoplasm disease, epstein-barr virus, jak inhibitor, primary myelofibrosis

## Abstract

Primary myelofibrosis (PMF) is a rare myeloproliferative neoplasm characterized by elevated platelet counts and fibrous tissues in the bone marrow. The JAK1/2 inhibitor (JAKi), ruxolitinib, has demonstrated efficacy in reducing splenic size, alleviating myelofibrosis-related symptoms, and improving overall survival. While an increased risk of lymphoproliferative disease (LPD) is suggested in patients with PMF, particularly those treated with JAKi, the involvement of Epstein-Barr virus (EBV) in such cases remains poorly documented. Here, we present the case of a 69-year-old woman with PMF who developed multiple lymphadenopathies and elevated soluble interleukin-2 receptor (sIL-2R) levels. Ruxolitinib and steroid therapy improved the symptoms for a short period; however, the lymphadenopathies and ascites eventually worsened. A biopsy confirmed EBV-positive diffuse large B-cell lymphoma, but the patient died of severe tumor lysis syndrome. Additionally, we conducted a literature review on EBV-related LPD in patients with primary and secondary myelofibrosis. Our report and literature review shed light on the occurrence of EBV-related LPD in MF, especially in those treated with JAKi, emphasizing the need to consider lymphoma as a potential diagnosis and monitor the EBV-DNA viral load in patients displaying lymphadenopathies or increased sIL-2R levels.

## Introduction

Primary myelofibrosis (PMF) is a rare subtype of myeloproliferative neoplasm (MPN) characterized by a significant increase in platelets and fibrous tissues in the bone marrow [1], With an incidence of approximately one in 100,000 individuals, the precise etiology of PMF remains elusive. However, recent studies have revealed that approximately 60% of PMF patients harbor the gain of function JAK2V617F mutation, 25% have CALR mutations, and 10% have MPL mutations [1,2]. These genetic aberrations result in constant upregulation of the JAK-STAT pathway, leading to abnormal proliferation of megakaryocytes and fibrosis in the bone marrow. Based on the upregulation of the JAK-STAT pathway in PMF, ruxolitinib, a JAK1/2 inhibitor (JAKi), was approved by the FDA in 2011 and has entered clinical practice. It provides significant clinical benefits in patients with PMF by reducing spleen size, ameliorating debilitating myelofibrosis-related symptoms, and improving overall survival [3]. However, recent reports indicate lymphoma development in patients with PMF treated with JAKi. One study revealed that JAKi treatment in PMF was associated with a 16-fold increased risk of lymphoproliferative disorders (LPD) [4] while others reported no significant difference in the risk of lymphoma development [5,6].

Epstein-Barr virus (EBV), a member of the herpesvirus family, infects over 90% of adults [7]. Primarily transmitted through saliva and contact with infected individuals, EBV persists throughout the lifetime of the host and remains latent in the resting memory B cells of immunocompetent individuals [8]. However, in patients with impaired immunity, EBV can reactivate, leading to the proliferation of EBV-infected B lymphocytes. Impaired cytotoxic T cells fail to remove the infected B cells, resulting in the development of malignancies. Indeed, EBV has been linked to Burkitt lymphoma, Hodgkin lymphoma, and lymphoproliferative disorders in immunocompromised individuals [7,9].

However, the association between EBV infection and PMF-associated LPD has been poorly studied. In this report, we present the case of a 69-year-old woman diagnosed with EBV-positive diffuse large B-cell lymphoma (DLBCL) during PMF treatment with ruxolitinib. Additionally, we conducted a literature review of EBV-positive LPD in primary and secondary myelofibrosis cases.

## Case presentation

**Figure 1:**
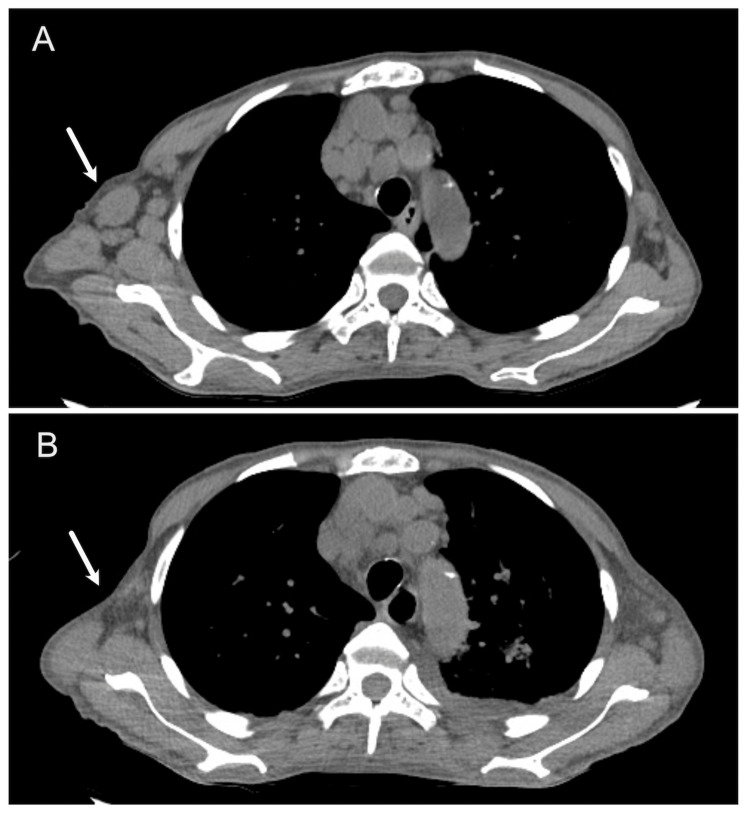
Results of CT scans (A) CT scans revealed systemic lymphadenopathy (arrow: axillary lymph nodes) at the time of admission. (B) CT scans after dexamethasone induction exhibited a mild reduction in the size of multiple lymph nodes.

A 69-year-old female with a medical history of PMF presented with a three-day history of lethargy and fever. A decade earlier, she had been referred to our hospital for thrombocytosis with a platelet count of 1,488,000/μL. The JAK2 V617F mutation was identified and bone marrow examination revealed hypercellularity of myeloid cells and megakaryocytes, accompanied by increased fibrosis in the absence of blasts. A diagnosis of low-grade PMF with JAK2 V617F mutation was established, leading to the initiation of anti-platelet therapy. Over the next several years, the disease gradually progressed. Platelet counts gradually decreased to less than 100,000/μL, and anemia worsened, necessitating frequent transfusions. Three months before the current presentation, the patient reported abdominal swelling and a weight gain of 3 kg within three weeks. Abdominal CT revealed aggravation of hepatosplenomegaly, ascites, and multiple lymphadenopathies. The soluble interleukin-2 receptor (sIL-2R) level was markedly elevated at 10,551 U/ml. Consequently, 5 mg of ruxolitinib was initiated, resulting in partial amelioration of fatigue. The introduction of 0.2 mg/kg (10 mg) of prednisone further improved her symptoms, including ascites and edema. However, one week before her current presentation, right lower back pain, followed by lethargy and fever, prompted her to seek hospital care. Upon presentation, her vital signs were normal except for fever. Systemic CT revealed worsened lymphadenopathy, and the sIL-2R level remained highly elevated at 21,496 U/ml. The serum EBV-DNA titer was markedly elevated to 6.69 log IU/ml, indicating the possibility of EBV-related LPD. The patient was admitted to our hospital. On the fourth day of hospitalization, a biopsy of the left inguinal lymph node was performed. Subsequently, on the seventh hospital day, prednisone 10 mg was changed to 4 mg of dexamethasone to address her persistent fever. Two days later, the patient developed severe dyspnea with low oxygen saturation, necessitating oxygen supplementation of 15-30L through a high-flow nasal cannula. Laboratory results revealed hyperkalemia with a level of 7.0 mEq/L, elevated urate level of 10.1 mg/dl, phosphate level of 11.4 mg/dl, and a lactate dehydrogenase (LDH) level of 5,365 U/L, indicating severe tumor lysis syndrome. Systemic CT revealed diffuse lung infiltrates indicative of pulmonary alveolar hemorrhage and bilateral pleural effusions, which were absent on admission, and a mild reduction in the size of the lymph nodes (Figure 1).

**Figure 2:**
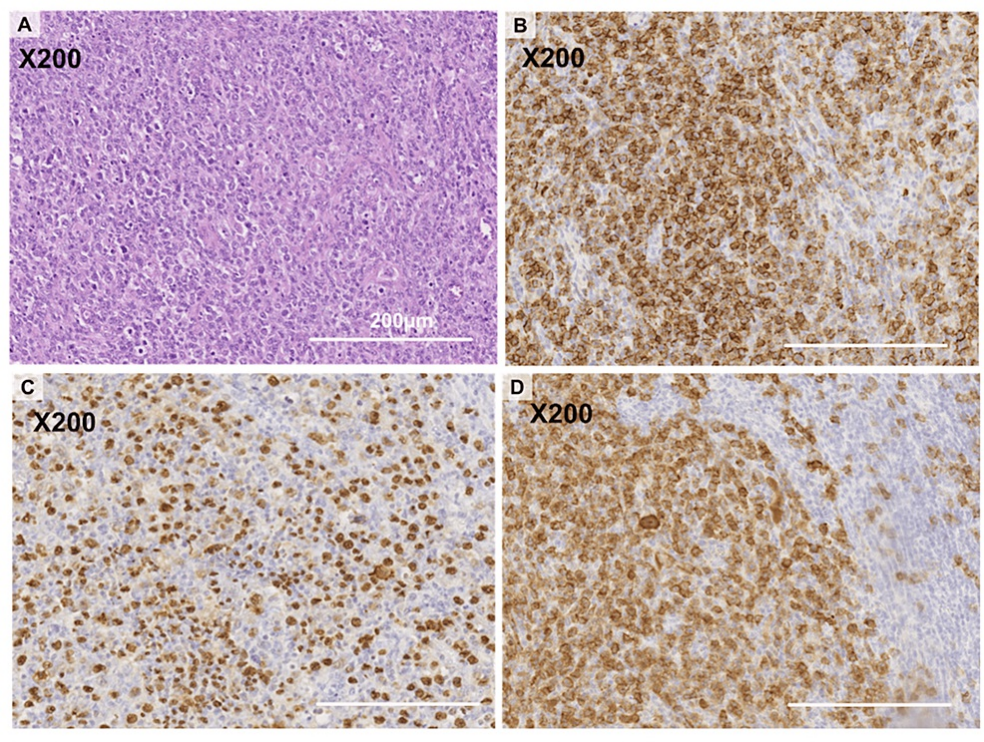
Pathological findings of the left inguinal lymph node (A) Hematoxylin & eosin (H & E) staining. (B) CD20 staining. (C) EBER staining. (D) CD30 staining.

The patient died the following day. The left inguinal lymph node biopsy confirmed multiple clusters of large cells expressing CD20, CD30, PAX5, and EBERs, and the histological type was consistent with that of EBV-positive DLBCL (Figure 2).

A subsequent autopsy revealed PMF with extramedullary hematopoiesis in the liver and spleen, alveolar hemorrhage, and diffuse alveolar damage. Furthermore, lymphadenopathies in the para-aortic, axillary, pelvic, peri-splenic, and supraclavicular regions were histologically proven to be EBV-positive DLBCL. 

## Discussion

We encountered a case of advanced-stage PMF that developed EBV-positive DLBCL and died of tumor lysis syndrome following dexamethasone initiation. The patient had already exhibited multiple lymphadenopathies and ascites when treatment with ruxolitinib was initiated. Interestingly, one study identified the immunoglobulin rearrangements in the bone marrow of approximately 15 % of PMF patients, indicating the presence of clonal B cells [4]. Thus, it is possible that our patient already had a B-cell clone that expanded during ruxolitinib treatment.

Given the central role of the JAK-STAT pathway in cytokine activation and signaling in immune cells, ruxolitinib, a JAKi, is known for its immunosuppressive effects, potentially leading to opportunistic infections and reactivation of latent viruses [10]. Ruxolitinib has been shown to increase the likelihood of EBV reactivation by 2.6-fold [11]. Furthermore, a number of case reports highlight the development of EBV-LPD during ruxolitinib treatment [12-15].

Regarding PMF, there is an ongoing debate about whether JAKi treatment increases the risk of lymphoproliferative disorders. One study revealed that JAKi treatment in myelofibrosis was associated with a 16-fold increased risk of aggressive B-cell lymphoma [4] while others reported no significant difference in the risk of lymphoma development between patients who received prior JAKi treatment and those who did not [5,6]. However, in previous studies, the association with EBV has been poorly documented.

To explore the potential connection, we conducted a literature review on EBV-related LPD occurring in myelofibrosis patients and identified only four cases, including our own (Table 1). On the other hand, some cases of EBV-negative LPD developed during JAKi therapy [4].

**Table 1:** Cases of EBV-positive lymphoproliferative disorder in patients with myelofibrosis JAKi: JAK inhibition, PMF: primary myelofibrosis, LPD: lymphoproliferative disorder, IHC: immunohistochemistry, PV: polycythemia vera, PCNSL: primary CNS lymphoma, NR: not reported, RTX: rituximab, ET: essential thrombocytosis, CHL: classical Hodgkin lymphoma, PVAG: prednisone, vinblastine, doxorubicin, gemcitabine, DEX: dexamethasone, PEG-IFN2α: pegylated interferon2α, DLBCL: diffuse large B-cell lymphoma

Patient No.	Age	Sex	Disease	JAK2V617F mutation	Treament before JAKi	JAK 1/2 inhibitor	LPD onset from JAKi	LPD type	LPD IHC	LPD treatment	Outcome	Ref.
1	70	M	PMF	positive	None	Ruxolitinib	30 months	DLBCL	MYC+ BCL-6+ P53+	R-CHOP	Death (2 yrs)	[4］
2	57	F	post PV MF	positive	Hydroxyurea, anagrelide PEG-IFN2α, splenectomy	Ruxolitinib	9 weeks	PCNSL	NR	Radiotherapy (24Gy12fr) RTX, Temozolomide	Death (5 wks)	[13］
3	56	M	post ET MF	negative (CALR mut)	NR	Ruxolitinib	14 months	CHL	CD30+ PAX5+ CD79a+ EBER+ CD20dim CD15-	Ruxolinitib cessation, RTX, PVAG	PR	[15］
4	69	F	PMF	positive	None	Ruxolitinib	3 months	DLBCL	CD20+CD30+ PAX5+ EBER+	DEX	Death (1 wk)	Our case

All patients received ruxolitinib treatment, suggesting a possible link between the immunosuppressive effects of JAKi and the development of EBV-related B-cell LPD. The onset of LPD after the initiation of JAKi treatment varied from 2 to 30 months. Despite the scarcity of PMF cases and the relatively recent introduction of ruxolitinib, which potentially hinders the establishment of a unified consensus, our literature review suggests a risk of EBV-LPD development during JAKi treatment.

However, some cases of EBV-negative LPD develop during JAKi therapy [4]. Further accumulation of cases is crucial to elucidate the manifestations and characteristics of EBV-related LPD in patients with PMF.

In cases in which lymphadenopathy or an increase in sIL-2R levels are observed in PMF, there is a rare but potential risk of concomitant malignant lymphoma, including EBV-related LPD. The prognosis can be dismal; thus, further investigations, such as biopsies, should be performed immediately before initiating treatment. In EBV-related LPD, the serum EBV-DNA titer serves as a marker for the disease [9]; thus, monitoring the EBV-DNA viral load can be useful in evaluating EBV association. 

## Conclusions

In patients with MF who display lymphadenopathies or elevated sIL-2R levels, there is a rare, albeit potential, risk of concurrent lymphoma. Clinicians should be aware of the association between EBV-LPD and PMF, especially with JAKi therapy. Therefore, thorough investigations, including biopsies, are warranted.
